# F-18 FP-CIT PET in Multiple System Atrophy of the Cerebellar Type: Additional Role in Treatment

**DOI:** 10.1155/2017/8598705

**Published:** 2017-11-29

**Authors:** Young Jin Jeong, Sang-Myung Cheon, Do-Young Kang, Jae Woo Kim

**Affiliations:** ^1^Department of Nuclear Medicine, Dong-A University Hospital, Dong-A University College of Medicine, Busan, Republic of Korea; ^2^Institute of Convergence Bio-Health, Dong-A University, Busan, Republic of Korea; ^3^Department of Neurology, Dong-A University Hospital, Dong-A University College of Medicine, Busan, Republic of Korea

## Abstract

We evaluated the difference in the status of dopamine transporters (DATs) depending on Parkinsonism, cerebellar, and autonomic features using F-18 FP-CIT positron emission tomography (PET) in multiple system atrophy with cerebellar ataxia (MSA-C). We also assessed whether the DAT PET could be useful in the management of MSA-C. Forty-nine patients who were clinically diagnosed as possible to probable MSA-C were included. Based on the F-18 FP-CIT PET results, patients were classified into normal (*n* = 25) and abnormal (*n* = 24) scan groups. There were statistically significant differences in rigidity, bradykinesia, postural instability, asymmetry, and specific uptake ratio (SUR) between the two groups but no significant differences in tremor and cerebellar/autonomic symptoms. Dopaminergic medications were administered to 22 patients. All seven patients with normal scans showed no change, while 10 of the 15 patients with abnormal scans showed clinical improvement. There was a trend of a negative correlation between levodopa equivalent dose and SUR, but it was not statistically significant. DAT imaging, such as F-18 FP-CIT PET, may be useful in predicting the response to dopaminergic medication regardless of cerebellar/autonomic symptoms in MSA-C. In addition to being used for the diagnosis of the disease, it may be used as a treatment decision index.

## 1. Introduction

Multiple system atrophy (MSA) is an adult-onset, sporadic neurodegenerative disorder pathologically characterized by prominent alpha-synuclein (a-Syn) inclusions with neuronal degeneration. Clinically, cardinal features of the disorder are Parkinsonism, cerebellar ataxia, autonomic failure, and corticospinal tract dysfunction. There are two clinical subtypes depending on the predominant motor presentation: a Parkinsonian variant reflecting underlying nigrostriatal degeneration (MSA-P) and a cerebellar variant associated with cerebellar ataxia (MSA-C) [1].

Although cerebellar symptoms are a major feature of MSA-C, Parkinsonian features are also observed [2]. This is supported by a pathologic study that revealed that a-Syn involvement and neuronal loss occurred not only in the cerebellum, pons, and olives but also in the striatum in MSA-C [3]. This nigrostriatal degeneration of MSA-C can also be visualized in imaging studies; it has been reported that striatal dopamine transporter (DAT) is reduced to various degrees in DAT imaging, such as F-18 fluorinated-N-3-fluoropropyl-2-b-carboxymethoxy-3-b-(4-iodophenyl)nortropane (FP-CIT) positron emission tomography (PET) [4–7]. F-18 FP-CIT PET can be used to assess dopaminergic neuronal degeneration by evaluating the density of DATs. Because many motor disorders characterized by Parkinsonian features exhibit various degrees of degeneration of dopaminergic neurons, DAT imaging, such as F-18 FP-CIT PET, is currently being used clinically for the evaluation of these diseases [5, 6]. In MSA, F-18 FP-CIT PET is mainly investigated in MSA-P, in which nigrostriatal symptoms are predominant, and it is relatively less studied in MSA-C. As seen in the MSA-C diagnostic guideline [1], there have been many studies on the use of perfusion single photon emission computed tomography or F-18 fluorodeoxyglucose (FDG) PET in the diagnosis of the disease by evaluating the decrease in blood flow or glucose metabolism in the cerebellum or striatum [8–12]. In the F-18 FP-CIT PET studies, various levels of striatal dopamine receptors have been reported [4–7], but the usefulness of the PET scan in disease evaluation has not yet been established in MSA-C.

Clinically, there is no cure for MSA, and management of MSA in focused on symptomatic relief [13]. Parkinsonism, cerebellar, and autonomic symptoms should be treated judiciously [2]. Dopaminergic medications for the treatment of Parkinsonism can often induce dyskinesia or aggravate autonomic symptoms, especially orthostatic hypotension [2]. This sometimes makes it difficult for neurologists to decide on medicines for Parkinsonism, but there are no indicators to support the determination of drug administration other than clinical judgments based on neurological examinations. In particular, cerebellar ataxia of MSA-C can make it hard for clinicians to evaluate Parkinsonism. Therefore, objective indicators of the status of Parkinsonism may help them make treatment decisions. Previous studies on idiopathic Parkinson's disease (IPD) have commonly reported that the DAT status of F-18 FP-CIT PET is associated with the degree of Parkinsonian symptoms [14–17]. Likewise, in MSA-C, F-18 FP-CIT PET can show the status of the presynaptic dopaminergic function, so it can be considered a marker of Parkinsonism in MSA-C. If so, F-18 FP-CIT PET may be helpful in the treatment as well as diagnosis of MSA-C.

In the present study, we evaluated the difference in the status of DAT depending on Parkinsonism, cerebellar, and autonomic features and assessed whether F-18 FP-CIT PET could be useful in the treatment of Parkinsonism in MSA-C.

## 2. Materials and Methods

### 2.1. Patients

Forty-nine patients with clinically possible or probable MSA-C (M : F = 30 : 19, 61.6 ± 6.5 yrs) were retrospectively enrolled in this study. Clinically, 21 patients had possible MSA-C (M : F = 13 : 8, 59.7 ± 6.3 yrs), and 28 patients had probable MSA-C (M : F = 17 : 11, 62.9 ± 6.4 yrs). The diagnosis of MSA-C was done by movement disorder specialists based on the current diagnostic criteria in patients with adult-onset (older than age of 40) progressive ataxia who had no relevant family history and no established acquired etiology of ataxia [1, 17]. All medical records were available, and the neurologists checked for motor disability, such as Parkinsonism (e.g., tremor, rigidity, bradykinesia, and postural instability), cerebellar features (e.g., gait ataxia, limb ataxia, cerebellar dysarthria, and cerebellar oculomotor dysfunction), and autonomic symptoms (e.g., orthostatic hypotension, urinary incontinence, and erectile dysfunction). The neurologists defined Parkinsonism as having definite bradykinesia and rigidity.

In patients taking dopaminergic medication for the treatment of Parkinsonism, doses of the drugs were investigated and expressed as levodopa equivalent doses (LEDs) [18]. Movement disorder specialists assessed the response to dopaminergic medication based on the clinical rating if there was a clinically meaningful improvement. Patients who took the medication for less than 3 months, discontinued the medication due to side effects, or were unable to confirm the response were excluded from the analysis.

Patients with Parkinson's disease, other atypical or secondary Parkinsonism, head trauma, stroke, dementia, or psychological disorders were excluded. In our normal database of F-18 FP-CIT PET, 10 healthy individuals who did not have any clinical symptoms related to Parkinsonism were selected. The Institutional Review Board of our hospital reviewed and approved the study protocol and informed consent form.

### 2.2. F-18 FP-CIT PET/CT and Image Analysis

All the F-18 FP-CIT PET/CT examinations were performed using a Discovery 710 PET/CT (GE Healthcare, Milwaukee, WI, USA) scanner. Patients were intravenously injected with 185 MBq F-18 FP-CIT and PET/CT acquisition was started 180 min after the radiotracer injection. F-18 FP-CIT was supplied by FutureChem in Republic of Korea. A helical CT scan was carried out with a rotation time of 0.5 s at 120 kVp and 100 mAs without an intravenous contrast agent. A PET scan followed immediately and was acquired for 10 min in the three-dimensional mode. All the images were acquired from the skull vertex to the skull base. The patients were allowed to continue their anti-Parkinson medication.

**Figure 1:**
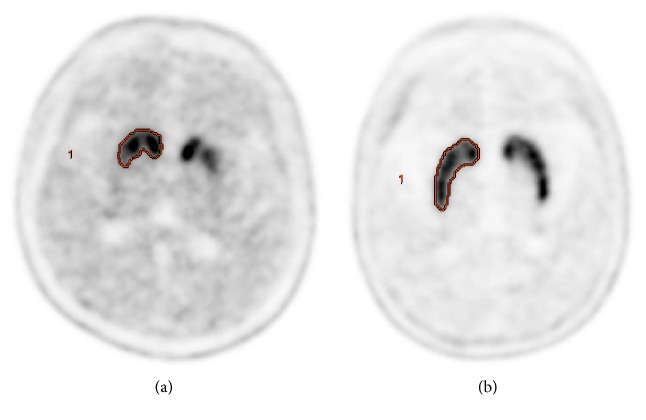
F-18 FP-CIT images of normal scan (a) and abnormal scan (b) groups. The gradient-based VOIs were automatically drawn on the striatum in the PET images.

Two experienced nuclear medicine physicians reviewed all the PET/CT images using a dedicated workstation with custom software (Advantage Workstation 5.0). The striatal volumetric analysis was done following a previous study [19]. To analyze the striatal functional volume, a semiautomatically delineated spherical volume-of-interest (VOI) was drawn over each of the two strata (Figure 1). The striatal target volume was segmented with custom software using a gradient-based method that detected the striatal margin based on a change in activity levels near the structure margin automatically [20]. We drew the VOI over the occipital lobe, and the value of the functional striatal volume multiplied by the occipital mean standardized uptake value (SUV mean) was considered nonspecific uptake of the striatum. Specific uptake ratios (SURs) were calculated for the target striatal VOI, and these values were defined as follows: mean standardized uptake value (mean SUV) of striatal VOI − mean SUV of occipital VOI/mean SUV of occipital VOI.

F-18 FP-CIT PET images were classified into normal and abnormal scans by visual and quantification analysis. First, the visual assessment was done using the morphology and density of the striatum and striatal asymmetry. Second, quantification analysis was performed based on the values of healthy subjects. The SUR cut-off value in the healthy subjects was 2.84. If the visual and quantification analyses showed the same results, the scan results were classified accordingly, while discordant cases were categorized by the agreement of two nuclear medicine physicians.

### 2.3. Statistical Analysis

The differences in patient characteristics and clinical symptoms between the normal and abnormal scan groups of F-18 FP-CIT PET were evaluated using the Mann–Whitney *U* test for continuous variables and the Chi-square test or Fisher's exact test for categorical variables. Fisher's exact test was performed to evaluate the response to dopaminergic medication according to the scan result. The relation between LED and various parameters was evaluated using Spearman's rho. Statistical analyses were performed using MedCalc software version 16.4 (MedCalc Software, Mariakerke, Belgium). Statistical significance was defined as *p* value < 0.05.

## 3. Results

### 3.1. Patients Characteristics and Difference between Normal and Abnormal Scan Groups (Table 1)

**Figure 2:**
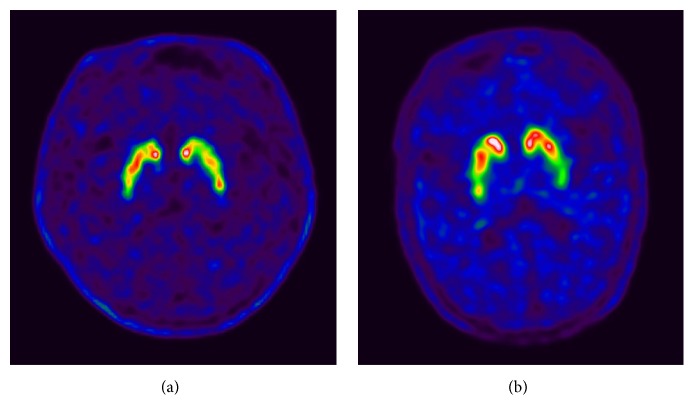
Two patients were normal in the quantitative analysis but abnormal in the visual assessment. One patient showed very heterogeneous uptake in both strata (a), and the other patient showed significantly decreased uptake in the tail portion of both putamens (b).

In F-18 FP-CIT PET, of all 49 MSA-C patients, 47 showed consistent findings in the visual and quantification analyses. Twenty-five patients were normal, and 22 were abnormal in both analyses consistently. The other two patients showed discordant results, which were quantitatively normal but visually abnormal. After checking again, one patient showed very heterogeneous uptake in both strata, and the other patient showed significantly decreased uptake in the tail portion of both putamens (Figure 2). Therefore, these two patients were placed in the abnormal scan group despite their normal quantification results. Finally, 25 (51.0%) patients were in the normal scan group, and 24 (49.0%) patients were in the abnormal scan group. There were no differences in age, sex, or disease duration between the two groups. Clinically confirmed Parkinsonism was more frequent in the abnormal scan group (32.0% versus 66.7%, *p* = 0.0163). Among Parkinsonian features, rigidity (*p* = 0.0004), bradykinesia (*p* = 0.0082), and postural instability (*p* = 0.0040) were significantly more common in the abnormal scan group than the normal scan group, but there was no significant difference in resting tremor (*p* = 0.3209). There was no significant difference between the two groups in the frequency of cerebellar features, such as gait ataxia (*p* = 1.0000), limb ataxia (*p* = 1.0000), cerebellar dysarthria (*p* = 1.0000), and cerebellar oculomotor dysfunction (*p* = 0.1383). There was also no significant difference between the two groups in the frequency of autonomic symptoms, such as orthostatic hypotension (*p* = 0.4624), urinary incontinence (*p* = 0.3255), and erectile dysfunction (*p* = 0.4425). In F-18 FP-CIT PET, the abnormal scan group showed significant asymmetry in the striatum (0.97 versus 0.88, *p* = 0.0006). SUR was also significantly lower in the abnormal scan group (3.98 versus 2.59, *p* < 0.0001).

### 3.2. Treatment Response of Dopaminergic Medication of Normal and Abnormal Scan Groups

**Figure 3:**
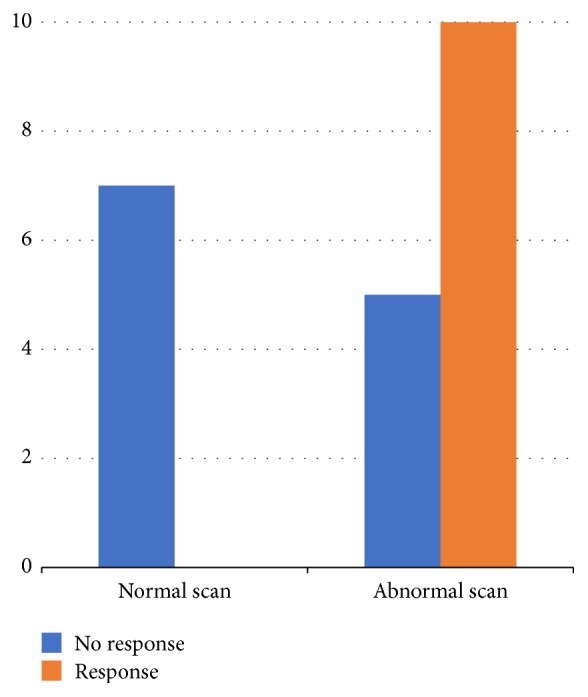
Bar chart showing responsiveness to dopaminergic medication in normal and abnormal scan groups. There was no response in all patients with normal scans and some response in the abnormal scan group.

Of the 49 patients, 39 had taken dopaminergic medication, including levodopa and dopamine agonists, according to the clinical judgment of neurologists, but 17 of these patients were excluded in the response analysis for the reasons mentioned above. Of the patients taking the drug, the remaining 22 patients were included in the analysis. Of the 22 patients taking medication, seven patients showed normal striatal uptake and 15 patients showed decreased striatal uptake in F-18 FP-CIT PET. After the chronic administration of dopaminergic medication (1.7 ± 0.9 years, 464 ± 200 mg/day in LED), 10 of 22 patients showed clinical improvement (45.5%). There was no response in all seven patients with normal striatal uptake. Ten of the 15 patients (66.7%) with abnormal striatal uptake showed a response to the medication (Figure 3). Fisher's exact test revealed a significant difference in the response rate between the normal and abnormal scan groups (*p* = 0.005).

### 3.3. Relationship between Dopaminergic Medication Dose and Striatal Uptake of F-18 FP-CIT PET

The mean LED was 464 ± 200 mg/day in the 22 patients who received dopaminergic medication. The mean LED was 386 ± 180 mg/day in the 12 patients who had no response to the drug and 488 ± 205 mg/day in the 10 patients who had a response to the drug, and there was no significant difference in the LED between the two groups (*p* = 0.2823). We examined the relationship of LED with the grade of bradykinesia, rigidity, and SUR in the 10 patients who were clinically responsive among the 22 patients who received dopaminergic medication. LED was not significantly correlated with grade of bradykinesia (*r* = −0.450, 95% confidence interval (CI) = −0.205–0.827, *p* = 0.1648) or rigidity (*r* = −0.426, 95% CI = −0.817–0.234, *p* = 0.1915). There was trend of a negative correlation between LED and SUR, but it was not statistically significant (*r* = −0.456, 95% CI = −0.844–0.243, *p* = 0.1848, Figure 4).

## 4. Discussion

In MSA, F-18 FP-CIT PET is widely used clinically for disease evaluation of MSA-P, because it represents the characteristic degeneration of presynaptic nigrostriatal dopaminergic nerves as in IPD. The clinical significance of F-18 FP-CIT PET has not yet been clearly established in MSA-C, but several studies have reported varying degrees of striatal uptake [4–7]. In the present study, of 49 patients, 25 (51.0%) were normal and 24 (49%) were abnormal in F-18 FP-CIT PET. The mean decline of SUR in the striatum was about 35% of that in normal subjects. Previous studies reported similar results of a reduction range between 21% and 79% [4, 7, 21]. Although it is generally known that nigrostriatal dopaminergic neurons are relatively uniformly reduced in MSA, the abnormal scan group of MSA-C in this study was asymmetrically reduced compared to normal subjects. These findings correlate well with a pathologic study indicating that neuronal loss of the nigrostriatal tract was heterogeneous according to the disease status [3], and a German multicenter study also reported that approximately 50% of MSA patients of their cohort revealed asymmetry of clinical symptoms [22]. Our results showed that striatal uptake of F-18 FP-CIT PET clearly reflected clinical Parkinsonian symptoms in MSA-C. Among the Parkinsonian features, the frequency of rigidity, bradykinesia, and postural instability and the severity of rigidity and bradykinesia were significantly higher in the abnormal scan group compared with the normal scan group. Resting tremor was not different between the two groups; this result could be explained by the fact that resting tremor is not directly related to the loss of nigral dopaminergic neurons [16]. This result was similar to those of previous studies on IPD [14–18]. Based on these results, F-18 FP-CIT PET also represents the nigrostriatal dopaminergic neuronal degeneration in MSA-C as in IPD.

However, in order for F-18 FP-CIT PET to have a significant clinical role in MSA-C, the results of the study should not be affected by motor dysfunction due to cerebellar or autonomic dysfunction. Our study showed that regardless of the F-18 FP-CIT PET results, most patients had cerebellar dysfunction and there was no significant difference in cerebellar or autonomic symptoms between the normal and abnormal scan groups. These results suggest that nigrostriatal neuronal degeneration occurs independently from cerebellar and autonomic neuronal degeneration. A previous study reported similar results, which showed no correlation between striatal uptake in DAT SPECT and clinical cerebellar disability [21]. Pathologic studies supporting these results indicated that region-specific cell loss was reported in MSA [3, 23, 24]. In particular, in MSA-C, neuronal loss predominantly involves the olivopontocerebellar structure and frequently the nigrostriatal tract and autonomic nuclei [3, 13]. However, region-specific neuronal degeneration occurs independently [23, 24]. Therefore, F-18 FP-CIT can demonstrate the status of presynaptic nigrostriatal dopaminergic degeneration regardless of cerebellar or autonomic dysfunction.

One of the purposes of the present study was to determine whether F-18 FP-CIT PET has a role in the treatment of Parkinsonism in MSA-C. Currently, symptomatic treatment is only available for MSA-C, and the two main targets of symptomatic treatment are Parkinsonism and autonomic dysfunction [2, 13]. Although the response to dopaminergic medication is poor or transient, about half of patients with MSA-C respond to the medication [2]. To evaluate drug responsiveness, the dopaminergic medications should be given for 3 months at an escalating dose, but orthostatic hypotension can be aggravated by the medication and about half of the patients with drug treatment show dyskinesia [2, 13, 22]. These problems make neurologists hesitant to prescribe medication; thus, they need an indicator that can help them decide whether to prescribe the medicine. In this study, through a chart review by a neurologist, we evaluated 22 patients' responsiveness to dopaminergic medications. All seven patients (100%) with normal scans showed no clinical response, and 10 of the 15 patients (66.7%) with abnormal scans showed a clinical response to the medication. Normal findings in F-18 FP-CIT PET suggested no nigrostriatal denervation. Therefore, the drug seemed to have no effect on all seven patients in the normal group. Clinically, even if a suspicion of Parkinsonism in MSA-C patients may be normal in F-18 FP-CIT PET, this discrepancy could be explained by the difficulty of diagnosis due to the manifestation of various motor function abnormalities in MSA-C. In the 15 patients who showed abnormal PET scans, 10 patients had a response to the drug, but one-third of the patients had no response. We could not find any difference in clinical and imaging characteristics between the two groups of patients (data not shown). Pathologic and imaging studies have demonstrated that postsynaptic dopamine D2/3 receptor and presynaptic DAT are also decreased in MSA [3, 25, 26]. Because dopamine D2/3 receptor provides inhibitory motor control, the reduction of the receptor leads to a loss of motor control. Since the motor dysfunction in MSA-C is due to the combined effect of pre- and postsynaptic receptors of the dopaminergic nerve, it is difficult to correctly assess the degeneration of dopaminergic neurons with only the F-18 FP-CIT PET showing only presynaptic DAT. In the presence of severe dopamine D2/3 receptor decline, an effect of the dopaminergic drug is unlikely. In this study, the five patients with no response to the drug of the 15 patients with abnormal scans would be considered in this case, but accurate evaluation requires additional dopamine D2/3 receptor imaging. Based on these results, it can be suggested that if F-18 FP-CIT PET shows normal findings in MSA-C patients, it may not be necessary to administer dopaminergic medications because drug effects are unlikely. In addition, in patients with abnormal PET findings, dopaminergic medication may be considered.

We thought that the degree of DAT reduction in F-18 FP-CIT might be correlated with the need for the drug. Nissen et al. reported that although the correlation was not strong, the amount of dopaminergic drug required increased significantly with decreasing DAT uptake (*r* = −0.26, *p* = 0.0201) [27]. In this study, a trend of an inverse association between LED and SUR was shown, but it was not statistically significant (*r* = −0.456, *p* = 0.1848). The possible explanation was that, as mentioned above, the state of the nigrostriatal dopaminergic pathway was not completely evaluated in F-18 FP-CIT PET showing only the presynaptic DAT state. In addition, the neurologist considered the adverse effect of the drug and adjusted the dose according to the patient's response. However, there was a trend of a negative relationship. Thus, a further study with a large population is needed.

The present study had some limitations. We could not investigate responsiveness to dopaminergic medication quantitatively, because this study was performed retrospectively based on a chart review. In addition, for the analysis of the response to dopaminergic medication, we included only those patients whose drug effects were clearly marked on the chart as a result of clinical judgments by movement disorder specialists. Moreover, we did not consider the normal aging effect in the quantification analysis of F-18 FP-CIT PET. Previous studies reported that DAT ligand (e.g., FP-CIT) binding in the normal striatum decreased with age at a rate of 5.3–7.7% per decade [28, 29]. Therefore, age correction is recommended for accurate quantification. In this study, except for four patients in their 70s, all patients were in their 50s and 60s. Most of the normal controls were also in their 50s and 60s. Thus, we thought the aging effect on the quantification results of F-18 FP-CIT PET would not be significant. Finally, misdiagnosis of clinically probable or possible MSA-C could be possible because no post-mortem confirmation was available. However, the diagnostic criteria for MSA have a high diagnostic accuracy [30]. Also, the mean disease duration of the patients was about 3 years, and patients with other cerebellar and Parkinsonism-related disease were excluded from this study.

In conclusion, this study suggests that F-18 FP-CIT PET imaging may be useful in predicting the effect of dopaminergic medication regardless of cerebellar or autonomic symptoms in MSA-C. In addition to being used for the diagnosis of the disease, F-18 FP-CIT PET may be used as a treatment decision index.

## Figures and Tables

**Figure 4:**
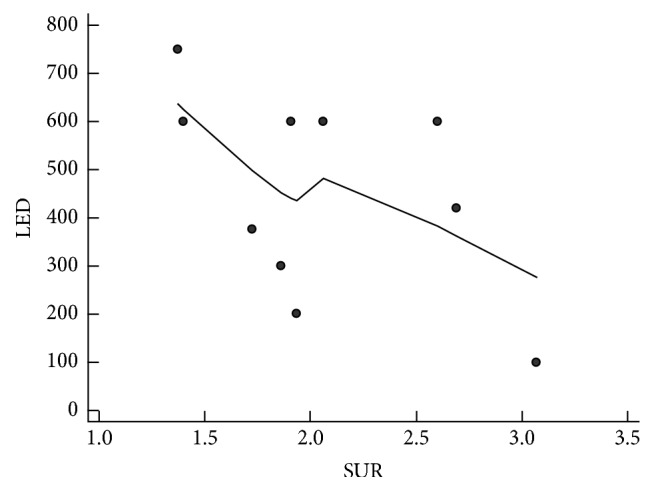
Correlation analysis between levodopa equivalent dose (LED) and specific uptake ratio (SUR) showing a trend of a negative, but not statistically significant, relationship (*r* = −0.456, *p* = 0.1848).

**Table 1:** Difference in characteristics of the patients between normal and abnormal scan groups.

Characteristics	Normal scan (*n* = 25)	Abnormal scan (*n* = 24)	*p*value^1^
Age (years)	61.7 ± 6.6	61.5 ± 6.5	0.9272
Sex (male : female)	18 : 7	12 : 12	0.0939
Disease duration (years)	3.1 ± 1.5	3.0 ± 1.6	0.8441
Parkinsonism	8 (32.0%)	16 (66.7%)	***0.0163***
Bradykinesia	14 (56.0%)	22 (91.7%)	***0.0082***
Bradykinesia (score)	1.0 ± 0.9	1.6 ± 0.8	***0.0142***
Rigidity	8 (32.0%)	20 (83.3%)	***0.0004***
Rigidity (score)	0.3 ± 0.5	0.9 ± 0.5	***0.0003***
Postural instability	17 (68.0%)	24 (100.0%)	***0.0040***
Resting tremor	4 (16.0%)	7 (29.2%)	0.3209
Cerebellar symptom			
Gait ataxia	25 (100.0%)	24 (100.0%)	1.0000
Limb ataxia	22 (88.0%)	22 (91.7%)	1.0000
Cerebellar dysarthria	22 (88.0%)	23 (95.8%)	1.0000
Oculomotor dysfunction	18 (72.0%)	22 (91.7%)	0.1383
Autonomic symptom			
Orthostatic hypotension	12 (48.0%)	9 (36.0%)	0.4624
Urinary incontinence	10 (40.0%)	13 (54.2%)	0.3255
Erectile dysfunction	10/18 (55.6%)	9/12 (75.0%)	0.4425
PET parameter			
Striatal asymmetry	0.97 ± 0.03	0.88 ± 0.10	***0.0006***
Striatal SUR	3.98 ± 0.58	2.59 ± 0.89	***<0.0001***

^1^ *p* value in bold, italic type indicates statistical significance. SUR: specific uptake ratio.
